# Rediscovery of a seventh sense: humans produce, perceive, and process ultrasound

**DOI:** 10.3389/fnins.2026.1732840

**Published:** 2026-05-20

**Authors:** Peter Schneider, Xu Feng, Ge Linbao, Li Jie, Mario Goncalves, Henry Johannes Greten

**Affiliations:** 1 Music Psychology and Brain Research Section, Department of Psychology, University of Graz, Graz, Austria; 2 Department of Neurology, Section of Biomagnetism, University of Heidelberg Medical School, Heidelberg, Germany; 3 Division of Neuroradiology, University of Heidelberg Medical School, Heidelberg, Germany; 4 Latvian Academy of Music, Riga, Latvia; 5 Heidelberg School of Chinese Medicine, Heidelberg, Germany; 6 Shanghai Qigong Research Institute, Shanghai, China; 7 Shanghai Academy of Traditional Chinese Medicine, Shanghai University of Traditional Chinese Medicine (SHUTCM), Shanghai, China; 8 IBA University of Cooperative Sciences, Darmstadt, Germany; 9 TCM Research Centre, Kinesiolab, Piaget Institute, Porto, Portugal

**Keywords:** auditory cortex, auditory evoked fields, pitch perception, qigong, ultrasound

## Abstract

**Introduction:**

We present novel evidence that humans are capable of producing, perceiving, and cortically processing ultrasound (US), extending the recognized limits of human auditory function. This previously unacknowledged sensory ability was identified in an expert practitioner of the traditional Chinese health exercise “The Six Healing Sounds.

**Methods and results:**

High-resolution recordings demonstrated deliberate vocal emissions reaching up to 35 kHz, with nearly one third of the acoustic energy residing in the ultrasonic range. Magnetoencephalography (MEG) revealed a distinct cortical response to these US components, characterized by an additional early N1 peak at 87 ms in the left auditory cortex—about 20–25 ms earlier than the conventional N1 response. Importantly, this US-specific component was entirely absent in a large cohort of 202 vocalists comprising 23 Chinese and 172 European trained singers and furthermore 7 experienced ‘Healing Sound’ singers, none of whom produced or perceived vocal US. Beyond cortical physiology, psychophysical testing demonstrated a unique perceptual strategy in the qigong practitioner: at very high frequencies his pitch perception shifted from spectral to fundamental mode, enabling him to reconstruct coherent auditory objects even when ultrasonic components were only weakly present.

**Discussion:**

This finding may reflect an exceptional adaptive neuroplastic mechanism integrating US into auditory and regulatory brain networks, akin to functional reorganizations observed in professional musicians. Taken together, these results provide the first demonstration in a human case study of intentional ultrasonic vocalization combined with cortical US processing in a human. The discovery not only challenges the traditional definition of auditory limits but also opens promising perspectives for the role of US in internal communication, vegetative regulation, and clinical neuromodulation. Low-intensity US stimulation may thus represent a novel therapeutic avenue for auditory and neurological disorders.

## Introduction

Ultrasound (US) is defined as sound waves with frequencies exceeding the upper limit of human hearing (>20 kHz). In contrast to humans, several animal species such as dogs, bats, dolphins, and certain rodents rely heavily on ultrasonic signals for navigation, communication, and social interaction, effectively employing US as an additional sensory modality (Au and Simmons, 2007). In these species, US functions almost as an idiomatic “seventh sense,” involving (1) the ability to generate ultrasonic vocalizations, (2) the capacity to perceive them as sensory stimuli, and (3) the integration of these signals within central nervous networks to mediate physiological and behavioral adaptations. Research in comparative bioacoustics has highlighted the evolutionary significance of such mechanisms, showing how ultrasonic cues can shape both neural organization and species-specific behavioral repertoires (Romani et al., 1982; Talavage et al., 2004).

By contrast, the human vocal apparatus—comprising the larynx, vocal folds, and supralaryngeal resonating cavities—has traditionally not been associated with sound production above the conventional audible range. Although human perception of ultrasonic frequencies has occasionally been demonstrated under artificial transmission conditions, particularly via bone conduction (Lenhardt et al., 1991), airborne ultrasonic vocalization and auditory cortical encoding of such signals have remained undocumented. Consequently, until recently, the medical and neuroscientific relevance of ultrasound in humans was restricted primarily to its technical applications, such as diagnostic sonography or micromassage-like stimulation of tissues (Bystritsky et al., 2011; Tyler et al., 2018). Nevertheless, converging evidence suggests that the human auditory system may not be strictly confined to the canonical hearing range. Early psychoacoustic studies already indicated perceptual sensitivity to frequencies slightly above 20 kHz (Pumphrey, 1950), and neurophysiological experiments have demonstrated that cortical responses can be modulated by ultrasonic stimulation when delivered via bone conduction (Zeng et al., 2000). More recent findings have expanded this perspective by showing that ultrasonic components are present in certain musical instruments (Petrosino and Canalis, 2016) and that targeted low-intensity ultrasound can modulate brain excitability, oscillatory activity, and behavior (Bystritsky and Korb, 2015; Hu et al., 2023). These insights raise the possibility that the human auditory system may possess underrecognized sensitivity to ultrasonic input, especially under conditions of training, plasticity, or unique vocalization strategies.

From a neuroplasticity perspective, parallels can be drawn with musical training, where experience-dependent modifications of auditory cortical maps have been robustly documented (Münte et al., 2002; Schneider et al., 2002, 2005). Professional musicians often exhibit enhanced synchrony and reorganized patterns of the auditory evoked P1–N1–P2 complex (Benner et al., 2023), reflecting adaptive changes in auditory pathways. Such findings suggest that, if ultrasonic perception or production were to occur in humans, similar plastic mechanisms could facilitate cortical integration of these signals. Taken together, current research indicates that while ultrasonic production and perception in humans have long been considered implausible, emerging evidence in psychoacoustics, neuromodulation, and musical neuroplasticity challenges this assumption. Demonstrating that humans are capable of intentionally producing, perceiving, and cortically processing US would therefore extend the recognized limits of auditory function and open new avenues for understanding both the evolutionary scope of human communication and the potential therapeutic applications of US in neuromodulation and auditory rehabilitation (Eggermont and Roberts, 2015; Seither-Preisler et al., 2014). Although human perception of US was previously unrecognized and thus its medical applications were considered limited to diagnostic purposes and micromassage-like effects on various bodily tissues, the accumulating evidence now suggests a far broader potential role in auditory neuroscience and therapeutic intervention.

## Materials and methods

### Participants

Case study with Q. M. (55 y, initials altered for confidentiality), a traditional Chinese Qigong master engaged in TCM Qigong and Taiji health research. For reasons of transparency, we note that Q. M. also served as a co-author of this manuscript. His dual role as participant and co-author was approved by the ethics committee (S-778/2018) and is reported here in line with current publishing standards. Furthermore, reference data for auditory tests, MEG, and in a subgroup of 67 also for MRI were obtained from 23 adult Chinese singers (37 ± 3 years) and 172 adult European singers (41 ± 4 years) and 7 experienced ‘Healing Sound’ singers (43 ± 6 years). All participants showed normal hearing levels (≤ 20 dB pure-tone thresholds) and no history of neurological disorders. Some of the participants in the two reference groups were taken from previous studies (Benner et al., 2023; Bücher et al., 2023). All participants were fully informed about the aims of the study and gave written informed consent before participation.

### Magnetencephalography (MEG)

Auditory evoked fields (AEFs) were recorded using a 122-channel whole-head MEG system with planar gradiometers (Neuromag-122; Hämäläinen et al., 1993). Stimuli comprised seven digitally sampled instrumental sounds (piano, guitar, flute, bass clarinet, trumpet, violin, and percussion) and five synthetic harmonic complex tones generated in MATLAB, as used in previous studies (Schneider et al., 2022, 2023). In an extended protocol, eight vocal and high-pass-filtered unvoiced speech sounds were additionally presented together with the instrumental sounds. Prior to measurement, four reference coils were attached to the participant’s head (left and right temples and left and right mastoid) with skin-friendly adhesive tapes. An electronic digitizing pen and a sensor on the forehead were first used to scan three points on the head surface that define the head coordinate system (nasion, right and left preauricular points). In addition, 32 other points on the head surface were digitized. For the MEG measurement, the participants were placed under the MEG dewar in a relaxed posture. To avoid an overlaying influence of task-specific changes in the auditory evoked responses, participants were measured without a task. The stimuli were presented binaurally via 90 cm plastic tubes through foam ear pieces placed in the ear canal and connected to small shielded transducers that were fixed in boxes next to the chair. Before MEG recording, the musical and vocal stimuli were calibrated at the output of the foam ear pieces, i.e., at the end of the 90 cm plastic tubes, using a Brüel & Kjaer artificial ear system (type 4,152, with an additional 2 cc coupler). The effective stimulation level at the point of acoustic delivery to the ear canal was adjusted to 70 ± 2 dB SPL. For the high-pass-filtered unvoiced speech sounds, playback level was increased at the transducer side by approximately 15 dB to compensate for high-frequency attenuation along the 90 cm plastic tubes.

The participants were instructed to listen attentively to the binaurally presented sounds in a relaxed state and to leave their eyes open while watching a silent movie. The position coils were then calibrated and their position relative to the MEG dewar was determined. Stimuli were presented in two continuous sequences of 17 min each to ensure a high signal-to-noise ratio. For each sequence, averaging across 1,200 stimulus presentations resulted in an expected noise reduction by a factor of √1,200 = 34.6, assuming uncorrelated noise. The first sequence included musical sounds and harmonic complex tones, whereas the second, extended sequence additionally included vocal sounds as well as high-pass-filtered unvoiced speech sounds. All stimuli had a duration of 500 ms and were presented with an interstimulus interval of 300–400 ms. To avoid clicks, identical 10-ms onset and offset ramps were applied to all stimuli using a Hann window.

The sensor waveforms were 330 Hz low-pass filtered and recorded using a sample rate of 1,000 Hz (filter range 0.00 (DC)-330 Hz). Data analysis was conducted with the BESA Research 6.0 software (MEGIS Software GmbH, Graefelfing, Germany). AEFs were calculated *post hoc* from the ongoing changes of the field distributions. Prior to averaging, data were inspected with the BESA Research Event-Related Field (ERF) Module to automatically exclude 3–7 noisy (bad) channels, about 10% of all epochs exceeding a gradient of 600 fT/cm × s, and amplitudes either exceeding 3,000 fT/cm or falling below 100 fT/cm. Signal strength was calculated relative to a 100 ms pre-stimulus baseline. The responses were collapsed into a grand average (on the average 1100 artifact-free epochs) in a 100 ms pre-stimulus to 400 ms post-stimulus time window. Based on a standard single-sphere head model, spatio-temporal source modelling was performed in normalized coordinates, independent of individual brain anatomy. Auditory cortical source activity was estimated using a bilateral equivalent-current-dipole model with one equivalent auditory cortical dipole in each hemisphere, as previously applied in auditory evoked field studies (Scherg et al., 1989; Scherg, 1990; Scherg and Picton, 1991; Schneider et al., 2005, 2022, 2023). In the present context, this bilateral two-dipole model refers to a left–right auditory cortical source model and not to the tangential–radial dipole model described by Scherg and von Cramon (1985, 1986). The P1–N1–P2 response complex was represented by one equivalent current dipole per hemisphere. Thus, the model was used to extract auditory cortical source waveforms of the P1–N1–P2 complex from the left and right hemispheres. Source modelling was performed on an individual basis prior to group-averaging of the source waveforms. Because head position under the MEG dewar varied between participants and stimulus conditions, source localizations and orientations were fitted individually using the same predefined fitting procedure. The fitting procedure was used to obtain stable left- and right-hemispheric auditory cortical source waveforms of the P1–N1–P2 complex and to extract N1 latencies and amplitudes in a comparable manner across participants. It was not intended to fit separate generators for P1, N1, and P2, nor to model the early N_1US_ component as an additional source. The fitting interval of the P1–N1–P2 complex was adjusted in four steps. First, the equivalent dipole in each hemisphere was temporarily converted into a regional source. This step was used to estimate the center of gravity of the auditory cortical response complex across the P1–P2 interval in a manner that was less dependent on a predefined dipole orientation. Second, the regional sources were converted back into single equivalent dipoles, yielding one representative left- and one representative right-hemispheric auditory cortical source waveform for further analysis. Third, dipole orientation was adjusted based on the clearly identifiable P1 deflection and its lower and upper half-side lobes. This constrained the source orientation to the main early auditory response while minimizing the influence of baseline noise or later response components. Fourth, the N1 and P2 portions of the response were evaluated within their corresponding half-sidelobe intervals while keeping the bilateral auditory cortical source model constant. N1 latencies and amplitudes were then extracted from the resulting left- and right-hemispheric source waveforms. This procedure was applied identically to Case Q. M. and to all reference participants. Importantly, the early N_1US_ component was not fitted as a separate source. It was consistently identified as an additional early deflection within the same left-hemispheric auditory cortical source waveform that also contained the conventional N_1norm_ component. The described procedure represents a specific variant of an established method for analyzing auditory evoked fields, similar to approaches applied in our previous studies (Benner et al., 2023; Schneider et al., 2022, 2023).

### Magnetic resonance imaging (MRI)

T1-weighted structural magnetic MRIs were performed in a subsample of 67 singers to investigate the anatomy of AC (Siemens, TrioTim, 3 Tesla). The data were acquired using 12 channel head coils and a standardized scanning protocol (MPRAGE), 176 DICOM slices, sagittal orientation; slice thickness 1 mm, field of view: 256 × 256; matrix size 128 K (16 Bit), repetition time (TR) = 1930 ms, echo time (TE) = 3.47 ms, flip angle 15. An individual approach of three-dimensional gray matter surface reconstruction of auditory subareas (HG, PT) was applied to account for individual morphology and gyrification patterns. For segmentation the Brain Voyager software QX 3.6 (Brain Innovation, B. V, Maastricht, NL) was used. All brain images were adjusted in contrast and in brightness, were precisely corrected for inhomogeneity and rotated in direction of the antero-posterior commissural line. The superior temporal plane including HG, aSTG and PT was segmented into sagittal MRI slices along the Sylvian fissure using the standard definition of the landmarks of AC (Schneider et al., 2005; Seither-Preisler et al., 2014; Serrallach et al., 2016; Benner et al., 2023).

### Auditory recordings, auditory discrimination tests and pitch perception preference

The auditory recordings have been made with a Sony PCM-D10 Portable High-Resolution Linear Recorder, Resolution 24 Bit, Sampling Rate 192 kHz, which reliably captures signals up to ~90 kHz without aliasing or harmonic distortion. Analysis of the frequency spectra have been made with the software VoceVista (Maass et al., 2004; Saus et al., 2025). The reported ultrasonic energy values refer to the relative spectral energy distribution within the recorded signal.

To measure the individual auditory discrimination and pitch perception profiles, the stimuli were presented binaurally using an RME Hammerfall DSP Multiface system and closed dynamic headphones (Sennheiser HAD 200) designed for high-quality hearing tests. These headphones provide a largely linear response up to ~16–18 kHz, but show a steep attenuation and increased harmonic distortion above this range. These headphones provide about 30 dB of passive attenuation in the frequency region of the stimuli used. The intensity was controlled not to exceed 70 dB SPL. Here, the auditory testing battery included the assessment of subjective pitch perception (Schneider et al., 2005).

The pitch perception preference test includes 144 different pairs of harmonic complex tones. Each pair consists of two consecutive tones (duration: 500 ms, 10 ms rise-fall time, interstimulus interval 250 ms). Each test tone includes two, three, or four adjacent harmonics, omitting the fundamental frequency. For each individual, an ‘index of pitch perception preference (ppp index)’ *δ* = (f_SP_-f_0_)/(f_SP_ + f_0_) was computed (f_SP_: number of perceived spectral pitches, f_0_ number of perceived fundamental pitches), see Schneider et al. (2005).

To gain a comprehensive understanding of auditory discrimination abilities and pitch perception profiles, we complemented our case data (Q. M.) with data from three reference groups (see above, 23 Chinese and 172 European adult singers and 7) ‘Healing Sound’ singers, partially taken from previous cross-sectional studies (Benner et al., 2023; Bücher et al., 2023). Auditory discrimination and pitch perception tests were analyzed using two-way ANOVAs, with discrimination thresholds for subjective pitch’ as the dependent variable.

## Results

### Ultrasonic vocalization in a human subject

**Figure 1:**
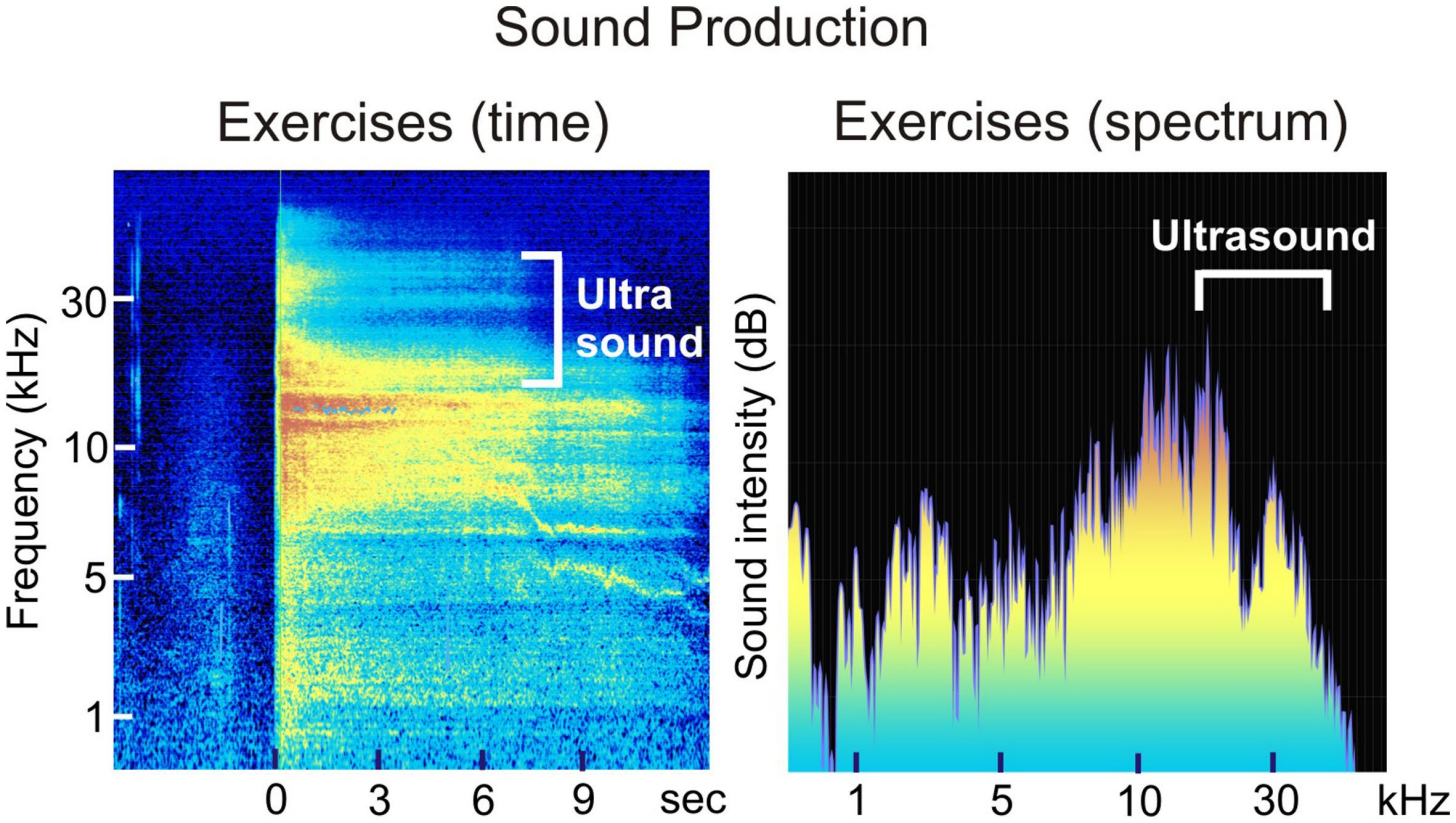
Sound production of one of the “Six Healing Sounds”; time course and frequency spectrum recorded from the expert Qigong practitioner Q. M.

We examined the vocal sound characteristics of an expert qigong practitioner performing traditional Chinese health exercises known as the “Six Healing Sounds” (Jahnke et al., 2010; Zhang et al., 2015), utilizing high-resolution audio recordings that captured ultrasonic frequencies (US). Qigong is a system of traditional Chinese health exercises combining breathing, movement, and vocalization. The specific style practiced by our subject (‘The Six Healing Sounds’) emphasizes controlled exhalations with distinct vocal timbres, providing a unique context in which ultrasonic emissions may arise. Spectral analysis conducted with VoceVista software (Maass et al., 2004; Saus et al., 2025) revealed vocalizations extending up to 35 kHz, with approximately 30% of the emitted acoustic energy residing within the ultrasonic frequency range (Figure 1). To our knowledge, this phenomenon has not previously been reported. Our findings thus provide the first evidence of intentional ultrasonic sound production by the human voice, prompting further inquiry into whether such ultrasonic vocalizations may be associated with neurobiological correlates or regulatory processes, although at present it remains unclear whether such ultrasonic vocalizations reflect a functional adaptation or an incidental acoustic feature.

### Auditory cortical responses to ultrasound

If our trained Chinese qigong expert Q. M., a long-term practitioner of the traditional Chinese health exercise qigong, evidently and routinely generates sounds that systematically include ultrasonic (US) components, it is likely that this ultrasonic signal becomes integrated into the neural networks underlying his vocal communication and internal regulatory processes, and therefore should also be perceptible to him as a sensory stimulus.

**Figure 2:**
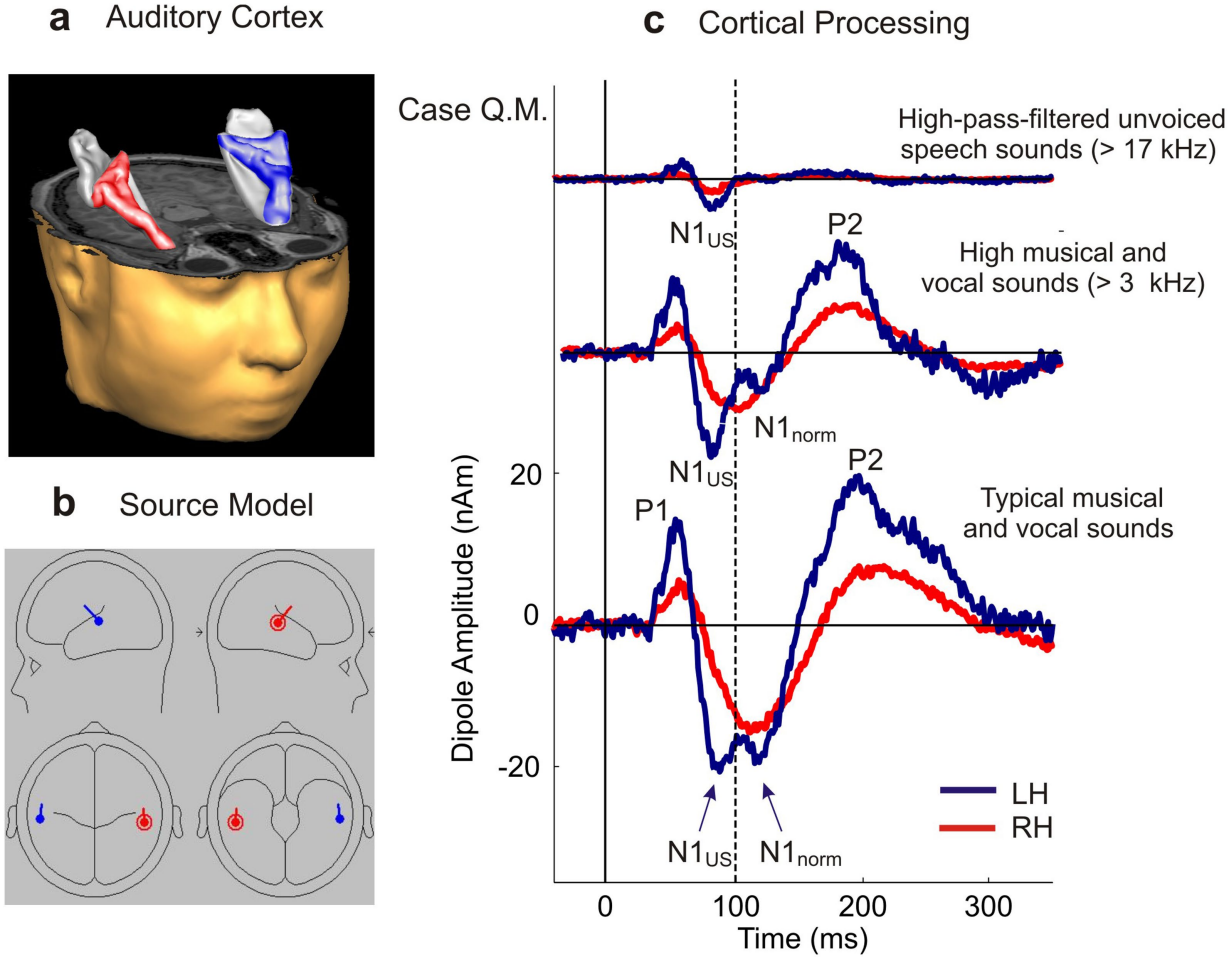
Sound processing. **(a,b)** Three-dimensional reconstruction of right and left AC and two-dipole model to extract the individual source waveforms (activation over time) in the regions of left (blue) and right (red) AC. **(c)** Auditory cortical responses of the expert Qigong practitioner Q. M. to typical unfiltered musical and vocal sounds (bottom trace), high-pass-filtered musical and vocal sounds (>3 kHz; middle trace), and high-pass-filtered unvoiced speech sounds (>17 kHz; top trace). The musical and vocal stimuli elicited both the normal N1 response component (N_1norm_) and an ultrasonic N1 response component (N_1US_) in the left hemisphere, while the >17 kHz unvoiced speech sounds still elicited the ultrasonic N_1US_ response.

To investigate this hypothesis, we employed magnetoencephalography (MEG) to record auditory evoked brain responses in the trained expert, as well as in two reference groups composed of 27 Chinese and 175 European experienced singers whose vocalizations have never been observed to contain US components. These reference groups were selected to control for possible cultural influences on auditory perception and furthermore to establish a robust baseline for comparative analyses. Auditory evoked responses including the P1, N1, and P2 components together were modeled by a single spatio-temporal dipole per hemisphere (Figures 2a,b; Scherg, 1990; Schneider et al., 2005, 2022, 2023). In 67 participants, also structural MRI scans were available, allowing precise coregistration with MEG data to confirm that sound processing consistently took place within the same cortical auditory regions, independent of potential cultural differences in size, shape and gyrification of AC. We hypothesized that the prominent N1 component, typically peaking around 100–125 ms after tone onset and recognized as a key marker for pitch processing (Bosnyak et al., 2004), may reflect frequency-specific individual differences in auditory perception.

Notably, distinct cortical responses to US were observed exclusively in our trained qigong practitioner, indicating that his AC is capable of detecting and processing US due to his specialized ability to produce it vocally. His auditory evoked fields revealed a characteristic split within the left-hemispheric auditory-evoked N1 component. This split response, observed in subject Q. M. during stimulation in MEG with high-quality musical and speech sounds containing only marginal residual US, consisted of a pronounced early peak at approximately 87 ms—associated with ultrahigh-frequency encoding—and a subsequent peak at approximately 110 ms, corresponding to the processing time of conventional frequencies typically found in music and speech (Figure 2c).

**Figure 3:**
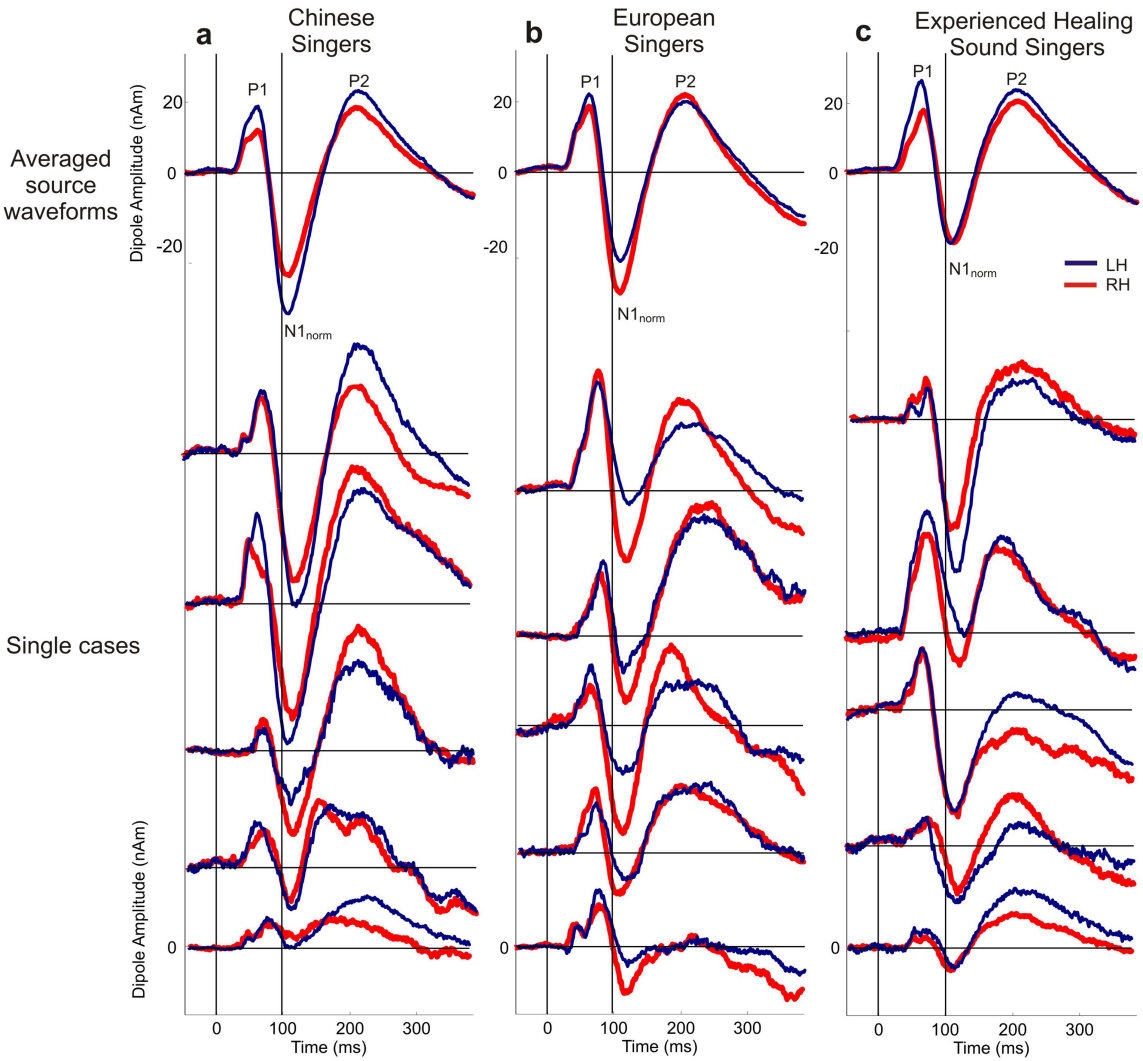
Auditory cortical responses of the three reference groups. Upper traces show group-averaged source waveforms; lower traces show representative individual source waveforms from each group. All reference groups and displayed individual cases show the expected normal N1 response (N_1norm_), but no left-hemispheric early N_1US_ component comparable to that observed in Q. M. in Figure 2. **(a)** Chinese singers, **(b)** European singers, and **(c)** experienced ‘Healing Sound’ singers; Q. M. is not included in these reference data.

Importantly, such distinctive US-specific components were consistently absent in the MEG responses of all singers from both reference groups, as clearly demonstrated by their response signals (averaged curves see Figure 3, top traces). Thus, this US-related early N1 component represents a unique neurophysiological signature exclusively identified in the experienced qigong master.

To clarify whether the early N_1US_ component could reflect an individual-level fluctuation rather than a stimulus-related auditory cortical response, we additionally inspected individual source waveforms from Chinese singers, European singers, and experienced healing sound singers. Figure 3 therefore shows both group-averaged source waveforms and representative individual source waveforms from each reference group. Although individual reference subjects showed the expected variability in response amplitude and waveform morphology, none displayed a comparable left-hemispheric double-peaked N1 pattern consisting of an early peak around 80–90 ms followed by a second N1 peak in the conventional latency range around 110 ms. The individual reference traces consistently showed a single dominant N_1norm_ component, whereas the early N_1US_ component was observed only in Q. M. Thus, the N_1US_ response is unlikely to be explained by the use of grand-averaged reference data alone or by a generic high-frequency fluctuation of individual baseline noise. Nevertheless, because the present evidence is based on a single case, this finding should be interpreted as an individual neurophysiological pattern requiring confirmation in future studies with additional cases and further controlled ultrasonic stimulation paradigms.

To furthermore exclude the possibility that the early N_1US_ component in Case Q. M. resulted from residual pre-stimulus baseline fluctuations, the MEG data were additionally reprocessed using the PCA-based correction procedure implemented in BESA Research. This correction reduced non–stimulus-locked baseline variability before recalculation of the auditory cortical source waveforms. After PCA correction, the pre-stimulus baseline was substantially stabilized, whereas the early left-hemispheric N_1US_ component at approximately 87 ms remained clearly present. The N_1US_ peak therefore cannot be explained by an ineffective 100 ms baseline correction or by a continuation of pre-stimulus noise activity. Instead, it represents a temporally distinct post-stimulus component preceding the conventional N_1norm_ response around 110 ms.

### Individual pitch perception profiles

As a third step, we established a subjective pitch perception profile designed to comprehensively assess individual variations in pitch perception abilities across the entire frequency spectrum. This paradigm has previously been established as a robust method to dissociate hemispheric contributions to pitch perception, particularly under conditions where the physical fundamental frequency is missing (Schneider et al., 2005; Seither-Preisler et al., 2014). Right auditory cortex activity has been linked to spectral pitch extraction, while left auditory cortex more strongly contributes to reconstructing the missing fundamental, thereby enabling a holistic representation of pitch (Schneider and Wengenroth, 2009). These hemispheric differences provide a well-validated framework for probing how listeners process both conventional and ultrahigh-frequency harmonic structures.

To accomplish this, the qigong practitioner, as well as the participants from the two reference groups, were presented with a wide range of harmonic complex tones from which the physical fundamental frequency systematically had been removed. We chose the missing-F0 paradigm rather than presenting simple F0 tones because it allows dissociating hemispheric strategies in pitch processing. While pure fundamental tones primarily engage tonotopic encoding of frequency, missing-F0 stimuli reveal whether the auditory system relies on right-hemispheric spectral analysis or left-hemispheric reconstruction of the absent F0 (Schneider et al., 2005; Schneider and Wengenroth, 2009). This makes the paradigm particularly suited to assess whether our subject’s perceptual strategy in the high-frequency and ultrasonic ranges involves a shift toward fundamental reconstruction. Thus, although F0 tones might provide a more direct measure of pitch thresholds, the missing-F0 task provides a more sensitive probe of hemispheric contributions to pitch perception, which was central to our research question.

Within this context, the test determines whether the spectral harmonics—those physically present in the stimulus—are predominantly perceived via the right hemisphere, or if otherwise the left hemisphere reconstructs the missing fundamental frequency.

**Figure 4:**
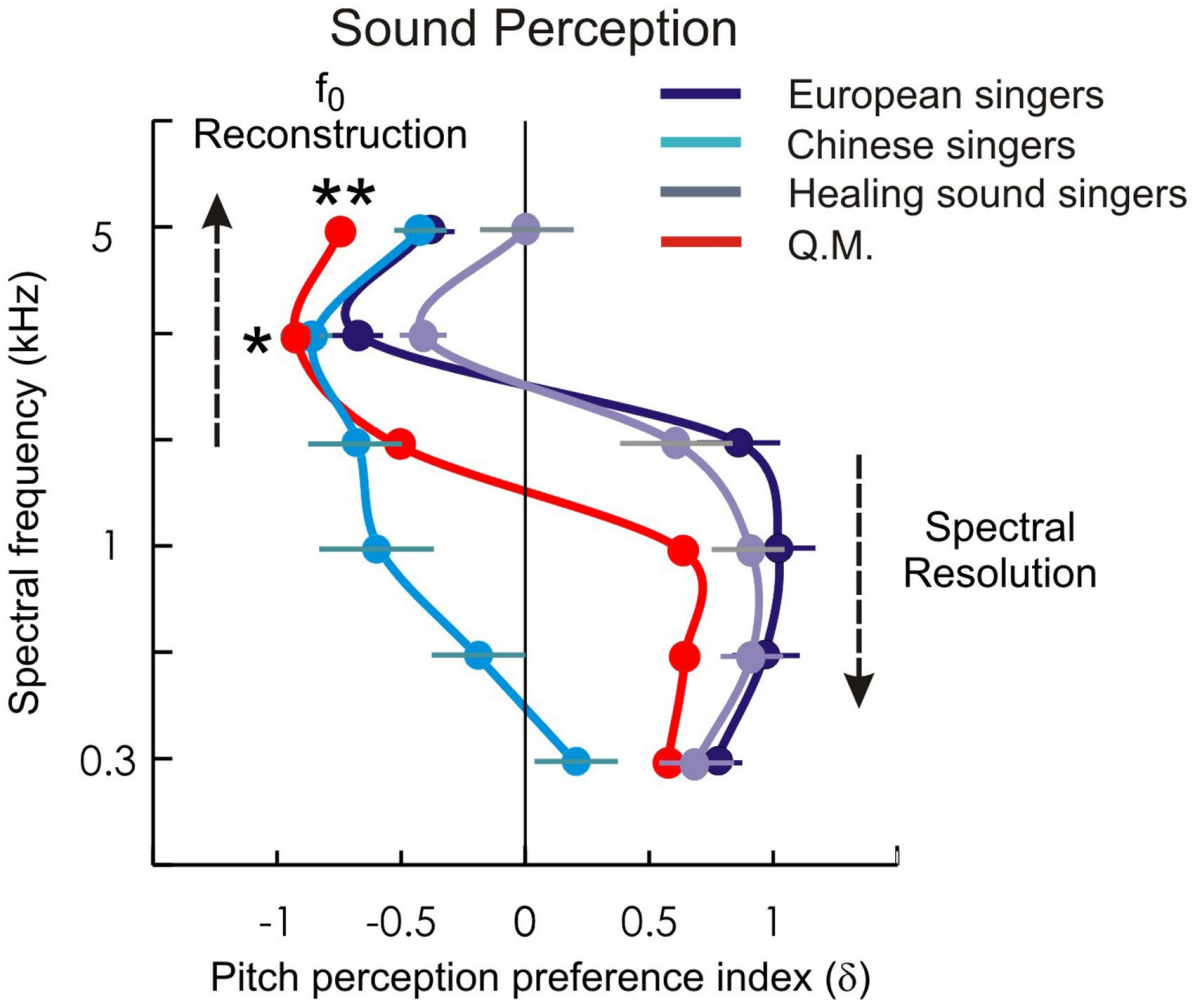
Sound perception. The subjective pitch perception preference profile of case Q. M. (red curve) is shown in comparison with the mean profiles of the three reference groups: Chinese singers, European singers, and experienced ‘Healing Sound’ singers (cyan, dark blue, and grey curves). At higher spectral frequencies (>3 kHz, *), Q. M. shows the strongest preference for fundamental-frequency (f0) pitch reconstruction across all groups, including Chinese and European singers as well as the other experienced ‘Healing Sound’ singers; this preference remains stable up to the highest frequencies tested (**). In contrast, pitch perception preference across the singer groups gradually shifts toward spectral pitch perception in the middle and lower frequency ranges (<2 kHz).

The resulting detailed and individualized pitch perception profiles effectively capture the relative contributions of spectral and fundamental pitch perception processes across both low and high frequency ranges [Figure 4; (Schneider et al., 2005; Schneider and Wengenroth, 2009), see methods section]. Interestingly, the qigong practitioner exhibited a remarkable shift in perceptual strategy at very high frequencies: while initially relying on spectral cues, his perception transitioned for high frequencies abruptly to a holistic mode dominated by fundamental pitch reconstruction (Figure 4). This shift allows him to perceive even the high-frequency sounds clearly as coherent auditory objects, effectively filling in missing components of the sound that are not or only marginally present in the stimulus.

Although Q. M.’s dual role as participant and co-author was approved by the ethics committee and is reported transparently, it may have introduced a potential source of response bias in the psychophysical testing. In particular, Q. M.’s expert knowledge of the study aims could, in principle, have influenced his response strategy or expectancy during the pitch perception preference task. However, the pitch perception preference test is a standardized psychophysical judgment paradigm rather than an open-ended self-report measure. Its outcome is derived from 144 randomized tone-pair judgments and has previously shown very high test–retest reliability over approximately six months (r = 0.96, *p* < 0.0001; Schneider et al., 2005). In addition, the paradigm has been linked to structural and functional asymmetries of the lateral Heschl’s gyrus, suggesting that it captures a stable perceptual-neurobiological trait. Nevertheless, expert knowledge of the study aims cannot be fully excluded as a potential influence on Q. M.’s behavioral responses. For this reason, the pitch perception preference data are interpreted as converging psychophysical evidence supporting the objective acoustic recordings and MEG findings.

### Chronotopic organization of the auditory responses

In auditory cortex, stimulus frequency is systematically related to response latency, such that higher frequencies evoke earlier N1 components – a phenomenon often described as chronotopic organization (Romani et al., 1982; Talavage et al., 2004; Benner et al., 2023). In our case study, the additional early N_1US_ response at ~87 ms falls within the latency range predicted by this gradient when extrapolated into the ultrasonic domain. This alignment strongly suggests that the early N_1US_ reflects immediate sensory encoding of ultrasonic input rather than unspecific cortical activity (Figure 2c).

The resemblance to missing-F0 processing is noteworthy. In classical missing-F0 paradigms, the auditory cortex reconstructs the percept of a fundamental pitch even when it is absent from the physical stimulus, by integrating harmonic cues across frequency channels (Schneider et al., 2005). Analogously, in Q. M., the presence of an additional early N1 component implies that the cortex is treating the ultrasonic input as a distinct auditory feature, despite its weak representation in the acoustic mixture. Since US is a strong natural component of his own voice, this experience may have led to the automatic formation of a unique neural fingerprint in the high-frequency range, enabling him to perceive coherent pitch objects even when US is only marginally present in the stimulus (Figure 4).

Thus, while the missing-F0 paradigm and ultrasonic responses differ in stimulus basis, both point to a capacity of auditory cortex to generate coherent percepts from incomplete or unconventional acoustic input. This analogy supports our interpretation that US is not only detected but also differentiated and integrated into the cortical auditory response of our subject.

## Discussion

The implications of this discovery extend beyond auditory perception. We hypothesized that, if US perception were consistently integrated into auditory cortical networks, it might engage functional properties similar to those observed in trained auditory systems, such as enhanced frequency mapping in musicians (Schneider et al., 2002, 2005). In the present case study, the additional early N1_US_ component suggests that the auditory cortex of the practitioner has adapted to encode ultrasonic input in a way that resembles training-induced plasticity.

To address whether the observed N_1US_ component reflects a general consequence of long-term practice of ‘Healing Sound’ exercises, we additionally analyzed a small reference group of experienced ‘Healing Sound’ singers. This group did not show a left-hemispheric early N_1US_ component comparable to that observed in Q. M.; instead, their source waveforms showed the conventional N_1norm_ response, similar to the Chinese and European singer groups. Likewise, although f0 reconstruction in the higher frequency range was present to some extent in singers, Q. M. showed the strongest and most sustained high-frequency f0 reconstruction profile across all groups. These findings indicate that the combination of N_1US_ and pronounced high-frequency f0 reconstruction was not a general characteristic of ‘Healing Sound’ practice in the present dataset. We therefore interpret Q. M.’s pattern as an exceptional single-case finding that may be compatible with an adaptive neuroplastic mechanism, but cannot yet be considered evidence for a practice-general effect. Future studies should examine larger cohorts of Qigong masters and long-term ‘Healing Sound’ practitioners, ideally combined with individual high-resolution acoustic recordings, to determine whether ultrasonic vocal components and their cortical correlates represent a reproducible functional adaptation or a rare case-specific phenomenon.

We therefore assessed the characteristic auditory evoked P1-N1-P2 complex (Seither-Preisler et al., 2014; Serrallach et al., 2016; Schneider et al., 2022, 2023) which serves as a functional fingerprint of AC (Benner et al., 2023) and can be used as a preliminary model of individualized response pattern profiles, potentially reflecting specific regulatory circuit functions of the human perceptual system. Trained musicians and singers typically exhibit a balanced pattern of these three subcomponents, reflecting training-induced plasticity of auditory cortex (Münte et al., 2002; Schneider et al., 2002; Bosnyak et al., 2004; Benner et al., 2023). The N1 component, located in the supratemporal sulcus, is particularly involved in chronotopic pitch mapping, having shorter latencies with increasing pitch height, typically at a gradient of 2–3 ms/octave (Romani et al., 1982; Talavage et al., 2004) ranging in adults between 100 and 125 ms. The superior temporal sulcus, known for its selective responsiveness to vocal sounds, plays a crucial role in this context, highlighting the brain’s specialized mechanisms for processing socially relevant auditory information such as speech, voices, and music (Belin et al., 2000; Zatorre et al., 2002, 2007; Vuust et al., 2022). Remarkably, our practitioner, who had no formal musical training besides his health exercises, exhibited functional patterns comparable to those of professional musicians, whose auditory cortical responses are well known to display training-induced reorganization and enhanced synchronization of the P1–N1–P2 complex (Münte et al., 2002; Schneider et al., 2002; Benner et al., 2023).

Most notably, alongside the typical expected response pattern peaking at ~110 ms (“N1_norm_” Benner et al., 2023, Schneider et al., 2023). Q. M. showed an additional early component at approximately 87 ms (N_1US_), resulting in a distinct left-hemispheric double-peaked N1 morphology. This early response occurred approximately 20–25 ms before the conventional N1 response and was not observed in the three reference groups, comprising Chinese singers, European singers, and experienced ‘Healing Sound’ singers (Table 1). Within the present single-case framework, this pattern supports the interpretation of an ultrasound-related cortical response in Q. M., rather than a general feature of singing expertise or

‘Healing Sound’ practice. This interpretation is compatible with the principle of chronotopic organization in the auditory cortex, according to which higher-frequency stimuli tend to elicit shorter-latency responses, particularly in pure-tone paradigms (Benner et al., 2023).

**Table 1:** Latencies and amplitudes of the auditory-evoked N1 component.

N1 parameter and hemisphere	European singers	Chinese singers	‘Healing Sound’ Singers	Case Q. M.
N1 latency RH (ms)	110.5 ± 0.8	107.9 ± 1.3	107.1 ± 2.2	*111.5 **106.3 ***87.9
N1 latency LH (ms)	109.2 ± 0.9	106.7 ± 1.2	105.4 ± 1.8	*87.4 (US)/112.3 (N) **86.5 (US)/114.6 (N) ***85.8 (US)
N1 Amplitude RH (nAm)	27.0 ± 2.5	21.5 ± 5.0	19.3 ± 6.1	*17.3 **11.8 ***2.2
N1 Amplitude LH (nAm)	21.1 ± 2.3	26.3 ± 4.7	19.1 ± 7.4	*19.5 (US)/18.7 (N) **15.2 (US)/5.6 (N) ***3.8 (US)

Reference-group values are reported as mean ± SEM; values for case Q. M. are single-subject values for the three stimulus conditions shown in Figure 2c: typical unfiltered musical and vocal sounds (*), high-pass-filtered musical and vocal sounds (**), and high-pass-filtered unvoiced speech sounds (***). For Q. M., the early ultrasound-related N1 component and the conventional N1 component are listed separately as N_1US_ and N_1norm_. US = ultrasound-related response; N = response in the normal auditory frequency range; LH = left hemisphere; RH = right hemisphere.

While our present findings were obtained using complex tones with a missing fundamental rather than simple tones, the observed early N1_US_ component in the ultrasonic range may reflect an analogous latency–frequency relationship. However, as the cited studies (Romani et al., 1982; Talavage et al., 2004; Bücher et al., 2023) primarily address tonotopic mapping and general response properties rather than latency shifts in missing-F0 conditions, the parallel should be regarded as a tentative analogy rather than direct evidence. Future work comparing pure-tone and missing-F0 paradigms will be necessary to confirm whether chronotopic principles extend to ultrasonic perception.

The extended stimulation protocol in Q. M. included high-pass-filtered musical and vocal sounds as well as high-pass-filtered unvoiced speech sounds, which strongly reduced conventional low- and mid-frequency acoustic components and emphasized ultrahigh-frequency spectral energy. Under these conditions, the early left-hemispheric N_1US_ component remained present at approximately 87 ms and became the dominant response in the high-pass-filtered unvoiced speech condition, whereas the conventional N_1norm_ component was no longer clearly expressed. This finding argues against the interpretation that the N_1US_ component merely reflects processing of conventional audible frequencies in complex musical sounds. Future studies using calibrated pure ultrasonic tones are required to determine whether the N_1US_ response can be selectively elicited by ultrasonic input alone.

An alternative explanation could be that the observed early N1 component reflects not genuine auditory processing of ultrasonic frequencies but secondary effects mediated by vibrotactile or somatosensory stimulation. Indeed, recent studies have shown that vibrotactile input can elicit cortical responses resembling auditory P1–N1 potentials (Huang et al., 2022). However, the present stimulation setup differed fundamentally from direct vibrotactile paradigms. Although the high-pass-filtered unvoiced speech sounds were driven at a higher transducer output level to compensate for attenuation in the 90 cm plastic tubes, the effective sound level was calibrated at the foam ear-piece output. The transducers themselves were mechanically decoupled from the participant’s head and were not in contact with the skull, mastoid, skin, or chair. Therefore, the level compensation ensured adequate airborne acoustic delivery at the ear canal, while the setup minimized direct mechanical vibration and bone-conducted stimulation. While such considerations highlight the need to carefully disentangle auditory from vibrotactile contributions, several aspects of our data argue against a purely tactile account. First, the stimuli were delivered via an air-conduction tube system rather than by direct mechanical contact with the head or body. Second, the distinct N_1US_ component was localized in the auditory cortex using MEG source modeling, with a chronotopic latency shift compatible with ultrahigh-frequency input. Finally, this component was absent in the reference groups tested with the same stimulation setup, including Chinese singers, European singers, and experienced ‘Healing Sound’ singers. Nevertheless, we acknowledge that auditory and somatosensory pathways may interact in processing extreme frequencies, and future studies should address this possible convergence by combining MEG/EEG with controlled auditory, bone-conduction, and tactile-only stimulation paradigms. Q. M’s exceptional ability to perceive US is further supported by his auditory perception profile, which demonstrates extended sensitivity to fundamental pitches exceeding the typical range of singer formants (~3–5 kHz) that characterize professional vocalists (Figure 4).

Together, these findings suggest that Q. M. possesses a unique capacity to transduce specifically high frequencies, including US into a lower audible range through cortical top-down processing mechanisms. A contemporary understanding of exercises of that type is that it serves as a vegetative biofeedback therapy calming down mind, emotions and inducing changes in physical activation. It is believed that the vegetative effects of these exercises can be conditioned by training (Matos et al., 2015). Thus, “The Six Healing Sounds” exercises may have induced specific adaptations in his AC, in the context of US perception. The observation that the deliberate use of the human voice in health exercises can trigger US-dependent responses opens up promising possibilities for therapeutic applications with sound and music, especially when combined with direct exposure and vibrations. Previous studies have shown that active listening can alleviate symptoms in conditions such as AD(H)D and dyslexia (Seither-Preisler et al., 2014; Serrallach et al., 2016). Furthermore, emerging evidence indicates that technical applications of US can modulate brain activity by influencing excitability and neural oscillations, with effects varying according to the frequency applied (Bystritsky and Korb, 2015). Notably, ultrasonic stimulation through technical devices and bone conduction has been utilized since the 1990s to influence brain functions and support the recovery from hearing impairments and tinnitus. Lenhardt et al. (1991) demonstrated that humans are capable of perceiving ultrasonic frequencies transmitted via bone conduction, thereby substantiating the potential of ultrasonic signals to influence auditory perception. Petrosino and Canalis (2016) expanded upon this understanding by identifying ultrasonic components naturally present within musical instruments, underscoring the brain’s capacity to process acoustic information beyond conventional hearing thresholds. Furthermore, clear evidence exists demonstrating that the human auditory system can detect frequencies exceeding previously established auditory limits, thus providing a physiological foundation for the therapeutic use of ultrasonic stimulation in treating hearing impairments and tinnitus (Pumphrey, 1950; Tyler et al., 2018; Bystritsky and Korb, 2015; Eggermont and Roberts, 2015; Zeng et al., 2000).

While our data demonstrate the capacity of a trained individual to produce and cortically process vocal ultrasound, they do not provide direct evidence for therapeutic efficacy or for an intrinsic human capacity to employ US for neuromodulation. Existing literature has documented beneficial effects of externally applied ultrasonic stimulation on auditory and neural functions (Lenhardt et al., 1991; Bystritsky and Korb, 2015; Tyler et al., 2018), and these reports highlight potential translational directions. However, such applications remain independent of the present findings, which are limited to a single case study and observational evidence. Nevertheless, our discovery broadens the understanding of human sensory perception and may ultimately redefine the role of US in health, neurologic and other functional disease. Low-intensity US, as demonstrated by Hu et al. (2023), can be applied to selectively modulate brain functions, offering a promising, targeted approach for various neurologic conditions.

## Data Availability

The raw data supporting the conclusions of this article will be made available by the authors, without undue reservation.
